# Cross-trait genetic analysis maps shared polygenic architecture between primary aldosteronism and blood pressure to adrenal cell states

**DOI:** 10.1186/s12967-026-08819-2

**Published:** 2026-08-13

**Authors:** Zhuolun Sun, Changying Jing, Yunxi Ji, Nicole Reisch, Tracy Ann Williams

**Affiliations:** Medizinische Klinik und Poliklinik IV, Klinikum der Universität München, Ludwig-Maximilians-Universität München, München, Germany

**Keywords:** Adrenal cortex, Blood pressure, Cross-trait genetic analysis, Endocrine hypertension, Polygenic architecture, Primary aldosteronism, Single-cell transcriptomics, Spatial transcriptomics

## Abstract

**Background:**

Primary aldosteronism (PA) is a major cause of endocrine hypertension and contributes substantially to blood pressure (BP)-related morbidity. Although aldosterone excess directly raises BP, the extent to which genetic susceptibility to PA overlaps with the broader polygenic architecture of BP remains unclear. We aimed to characterise the shared genetic architecture between PA and BP traits and to place shared genetic signals within adrenal tissue context.

**Methods:**

We analysed genetic overlap between PA and BP using two independent PA datasets from the French COMETE Network and the US Million Veteran Program, together with genome-wide association data for systolic BP, diastolic BP and pulse pressure. Analyses included genome-wide and regional genetic correlation, cross-trait meta-analysis to identify shared genetic signals and complementary gene-prioritisation approaches. Biological context was assessed using functional annotation, tissue-specific expression analyses and integration with single-cell and spatial transcriptomic datasets from human adrenal tissue.

**Results:**

PA showed significant positive genetic correlations with all three BP traits, with six genomic regions showing evidence of shared regional association. Cross-trait meta-analysis and co-localisation identified 143 independent shared signals associated with both PA and BP, with variant annotations indicating regulatory activity across adrenal, vascular, cardiac, adipose and neuroendocrine tissues. Gene-based analyses prioritised 43 candidate genes supported by convergent analytical approaches and implicated pathways related to vascular remodelling, extracellular matrix organisation, ion transport and endocrine regulation. Integration with adrenal single-cell and spatial transcriptomic data demonstrated that prioritised genes are expressed within defined adrenal cellular compartments, including aldosterone-producing populations. Among these candidates, SLC24A3 showed enriched expression in aldosterone-producing adenoma cells, with spatially heterogeneous expression associated with steroidogenic versus stress-adaptive transcriptional programmes. These findings support adrenal cell-state specificity of shared genetic signals and argue against a purely generic background BP-genetic explanation, without establishing causality for individual genes.

**Conclusions:**

PA and BP share a polygenic architecture that maps to adrenal endocrine cell states and vascular regulatory programmes within the intrinsic adrenal microenvironment. These findings link inherited BP-related variation to adrenal tissue biology and identify shared genetic signals, prioritised genes and cell states that may inform future studies of PA risk stratification, endocrine hypertension screening and cardiovascular risk prevention.

**Supplementary Information:**

The online version contains supplementary material available at 10.1186/s12967-026-08819-2.

## Background

Primary aldosteronism (PA) is the most common form of secondary hypertension, affecting 5–20% of patients with hypertension [1–3] and up to 30% of those with resistant hypertension, defined as blood pressure remaining uncontrolled despite treatment with at least three antihypertensive drug classes, including a diuretic [4, 5]. Beyond its classical effect on blood pressure (BP) through renal sodium retention, chronic aldosterone excess promotes cardiovascular injury through oxidative stress, vascular inflammation, endothelial dysfunction and myocardial fibrosis [6–10]. These effects contribute to excess cardiovascular morbidity and mortality in PA compared with essential hypertension at similar BP levels [10, 11]. Despite its prevalence and clinical impact, PA remains underdiagnosed and the extent to which its genetic susceptibility overlaps with the broader architecture of BP regulation remains unclear.

Genome-wide association studies (GWAS) have transformed understanding of BP as a complex trait. Large-scale studies of systolic BP (SBP), diastolic BP (DBP) and pulse pressure (PP) have identified hundreds of loci implicating renal sodium handling, vascular smooth muscle function, extracellular matrix remodelling and neurohormonal regulation [12–14]. In contrast, GWAS of PA have emerged only recently and remain constrained by smaller sample sizes, diagnostic complexity and phenotypic heterogeneity. Existing studies have identified susceptibility loci near *CASZ1*, *RXFP2* and *LSP1*, providing early insight into the genetic susceptibility to PA [15, 16]. Comparative analyses have suggested partial overlap between PA-associated variants and loci influencing BP or vascular traits [17] but whether PA and BP share a broader polygenic architecture has not been systematically defined.

This question is important because shared genetic signals could reflect several, non-mutually exclusive processes. They may indicate endocrine pathways influencing aldosterone production, vascular pathways contributing to BP variation or background genetic susceptibility to hypertension within PA cohorts. Distinguishing these possibilities is challenging from GWAS data alone, but integration with tissue-specific and spatially resolved transcriptomic data can help place shared loci within relevant biological contexts. In particular, adrenal single-cell and spatial transcriptomic datasets provide an opportunity to determine whether shared PA-BP risk genes are expressed within aldosterone-producing cell states and regional tissue niches.

Cross-trait genetic methods now enable systematic interrogation of shared genetic architecture across related diseases and quantitative traits [18, 19]. Genome-wide and regional genetic correlation analyses can estimate the extent and localisation of shared heritability, while cross-trait meta-analytical approaches can improve locus discovery by integrating association signals across genetically related phenotypes [20, 21]. Gene-prioritisation strategies that combine GWAS signals with functional genomic, transcriptomic and regulatory annotations can then help identify candidate genes, tissues and cell states through which shared risk may be biologically contextualised [22, 23]. Together, these approaches allow systematic examination of whether PA and BP traits share polygenic architecture and mapping of shared susceptibility to human adrenal biology.

In this study, we integrated two independent PA GWAS datasets, from the French COrtico et MEdullo-surrénale, les Tumeurs Endocrines Network (COMETE) and the US Million Veteran Program (MVP) [15, 24] with large-scale GWAS summary statistics for SBP, DBP and PP [13]. We aimed to quantify genome-wide and regional genetic correlations between PA and BP traits, identify shared genetic signals through cross-trait meta-analysis, prioritise candidate genes using convergent analytical approaches and map these genes to adrenal cell states using single-cell and spatial transcriptomic data from aldosterone-producing adenomas (APAs), a major cause of PA [25].

## Methods

### Study design and analytical framework

**Fig. 1:**
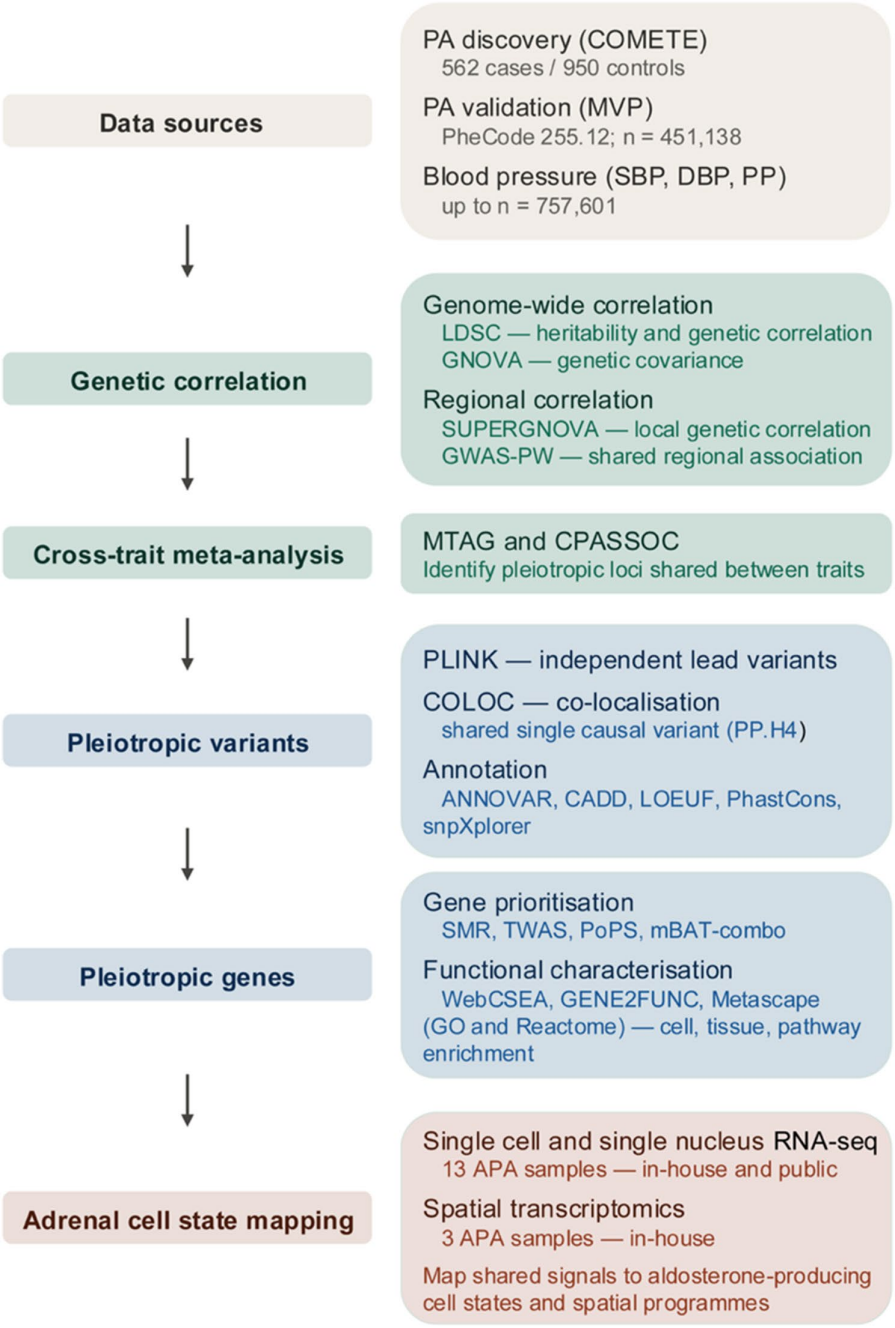
Analytical framework for mapping the shared genetic architecture between primary aldosteronism and blood pressure traits to adrenal cell states. Genome-wide association study (GWAS) summary statistics for primary aldosteronism (PA) from the COMETE cohort (562 cases and 950 controls) and the Million Veteran Program (MVP; PA cases identified by PheCode 255.12; *n* = 451,138) were integrated with large-scale GWAS data for systolic blood pressure (SBP), diastolic blood pressure (DBP) and pulse pressure (PP) from the ICBP and UK Biobank meta-analysis (up to *n* = 757,601). Genome-wide genetic correlation (LDSC, GNOVA) and regional genetic correlation (SUPERGNOVA, GWAS-PW) were used to quantify and localise shared polygenic architecture between PA and BP traits. Cross-trait meta-analysis (MTAG, CPASSOC) identified pleiotropic loci shared between traits, which were refined by clumping (PLINK), co-localisation (COLOC) and functional annotation (ANNOVAR, CADD, LOEUF, PhastCons, snpXplorer). Pleiotropic genes were prioritised by combining SMR, TWAS, PoPS and mBAT-combo, and characterised by cell-type (WebCSEA), tissue (GENE2FUNC) and pathway (Metascape; Gene Ontology and Reactome) enrichment. Prioritised genes were then mapped to adrenal single-cell and single-nucleus RNA sequencing data (13 aldosterone-producing adenoma [APA] samples; in-house and public) and spatial transcriptomic data (3 APA samples; in-house) to place shared genetic signals within cellular and spatial contexts in APA. APA, aldosterone-producing adenoma; CADD, Combined Annotation Dependent Depletion; COLOC, co-localisation analysis; COMETE, COrtico et MEdullo-surrénale, les Tumeurs Endocrines; CPASSOC, cross-phenotype association analysis; DBP, diastolic blood pressure; GNOVA, Genetic Covariance Analyser; GO, Gene Ontology; GWAS, genome-wide association study; GWAS-PW, Pairwise GWAS; ICBP, International Consortium for Blood Pressure; LDSC, Linkage Disequilibrium Score Regression; LOEUF, loss-of-function observed/expected upper bound fraction; mBAT-combo, Multi-Trait Gene-Based Association Test; MTAG, Multi-Trait Analysis of GWAS; MVP, Million Veteran Program; PA, primary aldosteronism; PoPS, Polygenic Priority Score; PP, pulse pressure; SBP, systolic blood pressure; SMR, Summary-data-based Mendelian Randomisation; TWAS, transcriptome-wide association study; UKB, UK Biobank

We systematically characterised the shared genetic architecture between PA and BP traits using European-ancestry GWAS summary statistics from two independent PA cohorts and three BP phenotypes (SBP, DBP and PP) [13, 15, 24]. We applied a stepwise analytical pipeline comprising genome-wide and regional genetic correlation analyses [19, 26–28], cross-trait meta-analysis [20, 21, 29], co-localisation [30] and gene-level integrative methods [31–34] to identify shared loci and prioritised candidate genes. These analyses were followed by functional, evolutionary, tissue-specific and cell-type annotation to assess the biological context of PA-BP genetic overlap [35–43]. To contextualise the genetic findings in adrenal tissue, we integrated single-cell, single-nucleus and spatial transcriptomic analyses of APA tissue [44–48]. Candidate-gene follow-up focused on *SLC24A3* and included in silico network perturbation [49], bulk transcriptomic validation [50–53] and siRNA knockdown. The overall study design is illustrated in Fig. 1.

### GWAS summary statistics

GWAS summary statistics for the PA discovery cohort were obtained from COMETE [15]. This dataset included 562 PA cases and 950 normotensive controls and is publicly available through the GWAS Catalog (GCST90129620). To support replication and assess consistency across datasets, we additionally used GWAS summary statistics from MVP, a large prospective cohort of United States veterans [24]. In the MVP cohort, PA cases (*N* = 451,138) were identified using PheCode 255.12, derived from ICD-9 code 255.10 and ICD-10 code E26.0 according to the standard PheWAS mapping framework (GCST90475695). For BP traits, we analysed GWAS summary statistics from the International Consortium for Blood Pressure (ICBP) and UK Biobank (UKB) meta-analysis, comprising up to 757,601 individuals of European ancestry [13]. Three BP phenotypes (SBP, DBP and PP) were selected to represent complementary haemodynamic components of BP regulation (SBP GWAS, GCST006624; DBP GWAS, GCST006627; PP GWAS, GCST006630). All genetic analyses were restricted to variants from participants of European ancestry to minimise population stratification and ensure consistency across PA and BP datasets.

### Genome-wide genetic correlation analysis

To quantify genome-wide genetic correlation between PA and BP traits, we applied linkage disequilibrium score regression (LDSC) [19] and genetic covariance analyser (GNOVA) [26]. These complementary methods estimate shared polygenic components between complex traits by evaluating covariance in genome-wide association signals while accounting for linkage disequilibrium (LD) structure. For LDSC, we used precomputed LD scores from the 1000 Genomes Project Phase 3 European reference panel, with the intercept left unconstrained. Analyses were restricted to HapMap3 SNPs shared across PA and BP summary statistics to reduce bias from poorly imputed or rare variants, and ambiguous SNPs were removed. The LDSC intercept was used to assess potential sample overlap and residual population stratification. GNOVA was applied as an independent approach to evaluate the robustness of genome-wide genetic covariance estimates. False discovery rate correction was applied using the Benjamini–Hochberg method, with q < 0.05 considered significant across PA–BP trait pairs. Genetic correlations supported by both LDSC and GNOVA were interpreted as evidence of reproducible genome-wide shared polygenic architecture.

### Regional genetic correlation analysis

To assess the regional distribution of PA–BP genetic overlap, we applied SUPERGNOVA to estimate local genetic correlations across predefined LD-independent genomic regions [27]. The genome was partitioned into independent LD blocks using the European reference panel described above. Statistical significance was determined using Benjamini–Hochberg false discovery rate correction, with q < 0.05 considered significant. To examine whether locally correlated regions were also consistent with shared association signals, we subsequently applied Pairwise GWAS (GWAS-PW), a Bayesian framework that estimates the posterior probability of shared genetic association within a genomic region [28]. Genomic regions with PPA3 ≥ 0.9 were considered to show strong evidence of shared regional association between PA and BP traits.

### Cross-trait meta-analysis

To identify variants associated with both PA and BP traits, we performed multi-trait analysis of GWAS (MTAG) and cross-phenotype association analysis (CPASSOC) [20, 21]. MTAG improves association testing for each trait by incorporating cross-trait genetic covariance, whereas CPASSOC evaluates cross-trait association using the SHet statistic, which accommodates heterogeneous effect directions and potential sample overlap. Because these methods capture complementary aspects of genetic sharing, we used a conservative intersection-based strategy to define robust cross-trait variants. SNPs reaching genome-wide significance (*P* < 5 × 10^− 8^) in both MTAG and CPASSOC were retained for downstream analyses. Independent lead variants were identified using PLINK v1.9 with an r² threshold < 0.02 and a 500-kb clumping window [29]. To further assess whether prioritised co-localised signals were supported by association evidence in the original PA GWAS datasets, we extracted SNP-level association statistics for each co-localised variant from the COMETE and MVP PA GWAS summary statistics. For PA GWAS files containing Z statistics only, two-sided P values were calculated from Z scores. Nominal PA-side support was defined as *P* < 0.05 in either PA GWAS dataset.

### Co-localisation analysis

To evaluate whether cross-trait association signals at selected loci were consistent with a shared underlying association, we performed co-localisation analysis using COLOC [30]. COLOC estimates posterior probabilities for five mutually exclusive hypotheses based on prior probabilities that a randomly selected SNP is associated with trait 1, trait 2 or both traits. Default priors were applied: p1 = 1 × 10^− 5^, p2 = 1 × 10^− 5^ and p12 = 1 × 10^− 5^. Loci with posterior probability for hypothesis 4 (PP.H4 ≥ 0.8), indicating support for a single shared causal variant, were considered to show evidence of co-localisation. Because co-localisation analyses are sensitive to locus definition, LD structure and available variant coverage, these results were interpreted as supportive evidence for shared genetic association rather than definitive evidence of mechanism.

### Functional and evolutionary annotation of cross-trait variants

Functional annotation of cross-trait variants was performed using ANNOVAR categories [35], CADD scores [36], LOEUF scores [37], PhastCons and PhyloP conservation scores [38] and snpXplorer [39]. These resources provided complementary information on variant consequence, predicted variant impact, evolutionary conservation, functional constraint and tissue-specific regulatory context. ANNOVAR categories and CADD scores were obtained through FUMA, with CADD > 12.37 used to identify variants with predicted functional relevance. LOEUF scores from gnomAD v4 were used to assess gene-level intolerance to loss-of-function variation, with LOEUF < 0.6 considered indicative of strong functional constraint. Evolutionary conservation was evaluated using PhastCons and PhyloP scores from VarSome [40], where higher PhastCons values and positive PhyloP scores indicate stronger cross-species conservation. snpXplorer was used to integrate expression quantitative trait loci (eQTL) and tissue-specific annotations to assess the transcriptional and tissue context of shared variants.

### Identification of PA-BP candidate genes

To prioritise candidate genes contributing to PA-BP genetic overlap, we applied four complementary approaches: Summary-data-based Mendelian Randomisation (SMR) [31], Transcriptome-Wide Association Study using the FUSION framework (TWAS/FUSION) [32], Polygenic Priority Score (PoPS) [33] and Multi-Trait Gene-Based Association Test (mBAT-combo) [34]. SMR and TWAS/FUSION integrate GWAS summary statistics with cis-eQTL data to identify genes whose genetically regulated expression is associated with the trait of interest. PoPS prioritises genes by integrating polygenic enrichment across biological pathways, gene expression patterns and protein-protein interaction networks. mBAT-combo aggregates multi-trait gene-level association statistics to improve detection of genes associated with multiple traits. Genes were considered credible PA-BP candidate genes if they met all the following criteria: (a) Bonferroni-corrected *P* < 0.05 in SMR, TWAS/FUSION and mBAT-combo; (b) HEIDI *P* > 0.01 in SMR; and (c) PoPS score > 0.8. These criteria were selected to prioritise genes supported by convergent evidence across complementary gene-prioritisation methods.

### Pathway enrichment analysis and clustering

Pathway enrichment analysis and enrichment clustering were performed using Metascape [41]. Hierarchical clustering was applied to enriched terms. Pairs of terms with a kappa score > 0.3 were considered part of the same cluster and each cluster was represented by its most statistically significant term.

### Tissue-specific enrichment analysis

Tissue-specific enrichment analysis was performed using the GENE2FUNC function in FUMA, based on two Genotype-Tissue Expression (GTEx) datasets covering 30 and 53 tissues [42]. Differentially expressed genes were identified in FUMA by performing two-sided t-tests for each tissue against all other tissues using log2-transformed, normalised expression values. Genes with Bonferroni-adjusted *P* < 0.05 and |log2 fold change| ≥ 0.58 were considered tissue-specific differentially expressed genes. Upregulated and downregulated gene sets were evaluated according to the sign of the t-statistic.

### Cell type-specific enrichment analysis

Cell type-specific enrichment analysis was performed using WebCSEA [43] which evaluates gene-set enrichment across tissue–cell type signatures derived from large-scale single-cell RNA sequencing datasets. Dai et al. [43] profiled more than 5.5 million cells from 111 tissues and 1,355 tissue–cell type signatures, removed low-expression genes and generated tissue-cell type-specific signatures using the t-statistic-based deTS method. For each cell type, genes within the top 5% of t-statistic scores were defined as tissue-cell type-specific markers. Fisher’s exact test was used to determine whether shared candidate genes for each PA–BP trait pair were enriched in these marker sets. Statistical significance was defined as Bonferroni-corrected *P* < 0.05.

### Single-cell and single-nucleus RNA sequencing analysis

Single-cell and single-nucleus RNA sequencing data were collected from two publicly available APA datasets comprising 11 APA samples in total (syn6023883, snRNA-seq, *n* = 8; GSE242404, scRNA-seq, *n* = 3) [44, 45], together with two additional APA samples generated in our previous study [46]. All datasets were processed and analysed using the Seurat R package. For quality control, cells or nuclei were excluded if they met any of the following criteria: fewer than 500 or more than 7,500 detected genes; fewer than 300 or more than 20,000 unique molecular identifiers; or mitochondrial RNA content exceeding 20% of total unique molecular identifiers. Potential doublets were identified and removed using DoubletFinder. After filtering, gene expression data were normalised using SCTransform, batch effects were corrected using Harmony, and dimensionality reduction, clustering and visualisation were performed using principal component analysis and Uniform Manifold Approximation and Projection. Cell-type annotations were assigned according to established canonical marker genes. To assess the expression of PA-BP candidate genes at the single-cell level, gene sets derived from the genetic analyses were used to calculate module scores using AddModuleScore.

### Spatial transcriptomic analysis

Spatial transcriptomic data from three formalin-fixed, paraffin-embedded APA samples generated in our previous study [46] were analysed using the Seurat package. Data were normalised using SCTransform, followed by dimensionality reduction with principal component analysis. Multiple samples were integrated using an anchor-based integration workflow, with integration anchors identified by canonical correlation analysis before clustering. Cluster annotation was guided by haematoxylin and eosin-stained sections together with cluster-specific marker gene expression. Spatial gene expression patterns were visualised using SpatialDimPlot and SpatialFeaturePlot.

### Spatial dependency analysis

To model spatially informed pathway relationships within APA tissue, we applied MISTy (Multiview Intercellular SpaTial modelling framework) to spatial transcriptomic data [47]. Heterogeneity analysis was performed using 12 cell states and pathways curated in the CancerSEA database, which capture conserved cellular states and stress-adaptive programmes relevant to tumour and tissue heterogeneity [48]. Pathway activity scores were calculated for each spatial spot based on CancerSEA gene signatures and used as inputs for spatial modelling. MISTy was used to decompose pathway dependencies across multiple spatial contexts, including IntraView, representing cell-intrinsic associations; JuxtaView, representing local neighbourhood associations; and ParaView, representing broader tissue-level context. This multiview approach enabled assessment of how pathway activities at a given spatial location were associated with pathway activities in surrounding regions across different spatial scales. To examine *SLC24A3*-associated spatial heterogeneity, APA spots with SCT-normalised *SLC24A3* expression greater than zero were classified as *SLC24A3*-positive, whereas those with zero expression were classified as *SLC24A3*-negative. Spatial association networks were then constructed to evaluate spatial dependencies between *SLC24A3*-defined APA regions and CancerSEA pathway activities across spatial views. These analyses were used to characterise spatial transcriptional associations and were not interpreted as evidence of direct functional interaction.

### In silico perturbation analysis of *SLC24A3*

To explore whether *SLC24A3* was connected to adrenal transcriptional networks in APA, we performed computational perturbation analysis using scTenifoldKnk, a single-cell RNA-seq-based framework for in silico gene perturbation [49]. *SLC24A3* perturbation was simulated by removing the *SLC24A3* node from the inferred gene regulatory network and estimating downstream changes in network structure. This approach was used to assess potential network-level dependencies associated with *SLC24A3* in APA cells, without assuming direct regulation of predefined downstream targets. Genes affected by simulated perturbation were ranked according to Z scores and associated *P* values derived from network perturbation statistics. The Z score reflects the magnitude and statistical support for altered regulatory connectivity following perturbation, rather than the direction of gene expression change. These analyses were considered exploratory and hypothesis-generating.

### Bulk transcriptomic analysis

Bulk RNA sequencing data from the adrenal zona glomerulosa layer of pigs were obtained from our previous study and used to assess expression patterns of PA-BP candidate genes under high-salt and low-salt dietary conditions [50]. These data provided an experimental context relevant to aldosterone regulation. In addition, three publicly available human adrenal bulk transcriptomic datasets (GSE8514, GSE60042 and GSE156931) were retrieved from the Gene Expression Omnibus database [51–53]. Together, these datasets comprised 20 non-tumoural adrenal cortex samples and 25 APA samples. These data were used to evaluate *SLC24A3* expression differences between APA and non-tumoural adrenal cortex and to assess the association between *SLC24A3* and *CYP11B2* expression across samples.

### Cell culture and siRNA transfection

HAC15 human adrenocortical cells were cultured at 37 °C with 5% CO₂ in DMEM/F-12 (1:1; Gibco) supplemented with 10% Cosmic Calf Serum (HyClone), 1 x insulin-transferrin-selenium, 1% antibiotic–antimycotic and 0.01% gentamicin. Prior to treatment, cells were serum-starved in DMEM/F-12 containing 0.1% Cosmic Calf Serum. Cells were transfected with 200 nM non-targeting control siRNA or *SLC24A3*-targeting siRNA using the Amaxa Cell Line Nucleofector Kit R with a Nucleofector II device (program X-005; Lonza), according to the manufacturer’s protocol. siNC#1 (negative control, 4390843) and si*SLC24A3* (4392420-s33028) were obtained from Thermo Fisher Scientific. Cells were harvested 48 h after transfection for RT-qPCR.

### RT-qPCR

RNA was extracted using the Maxwell RSC simplyRNA Cells Kit (Promega) and cDNA was synthesised using oligo(dT) primers and the GoScript Reverse Transcription System (Promega). Quantitative PCR was performed on a QuantStudio 5 Flex Real-Time PCR System (Thermo Fisher Scientific) using TaqMan Gene Expression Assays for *GAPDH* (Hs02786624_g1), *CYP11B2* (Hs01597732_m1) and *SLC24A3* (Hs00915003_m1). Gene expression was normalised to *GAPDH* and quantified using the 2^−ΔΔCt^ method.

## Results

### Genetic correlation reveals shared polygenic architecture between PA and BP traits

**Fig. 2:**
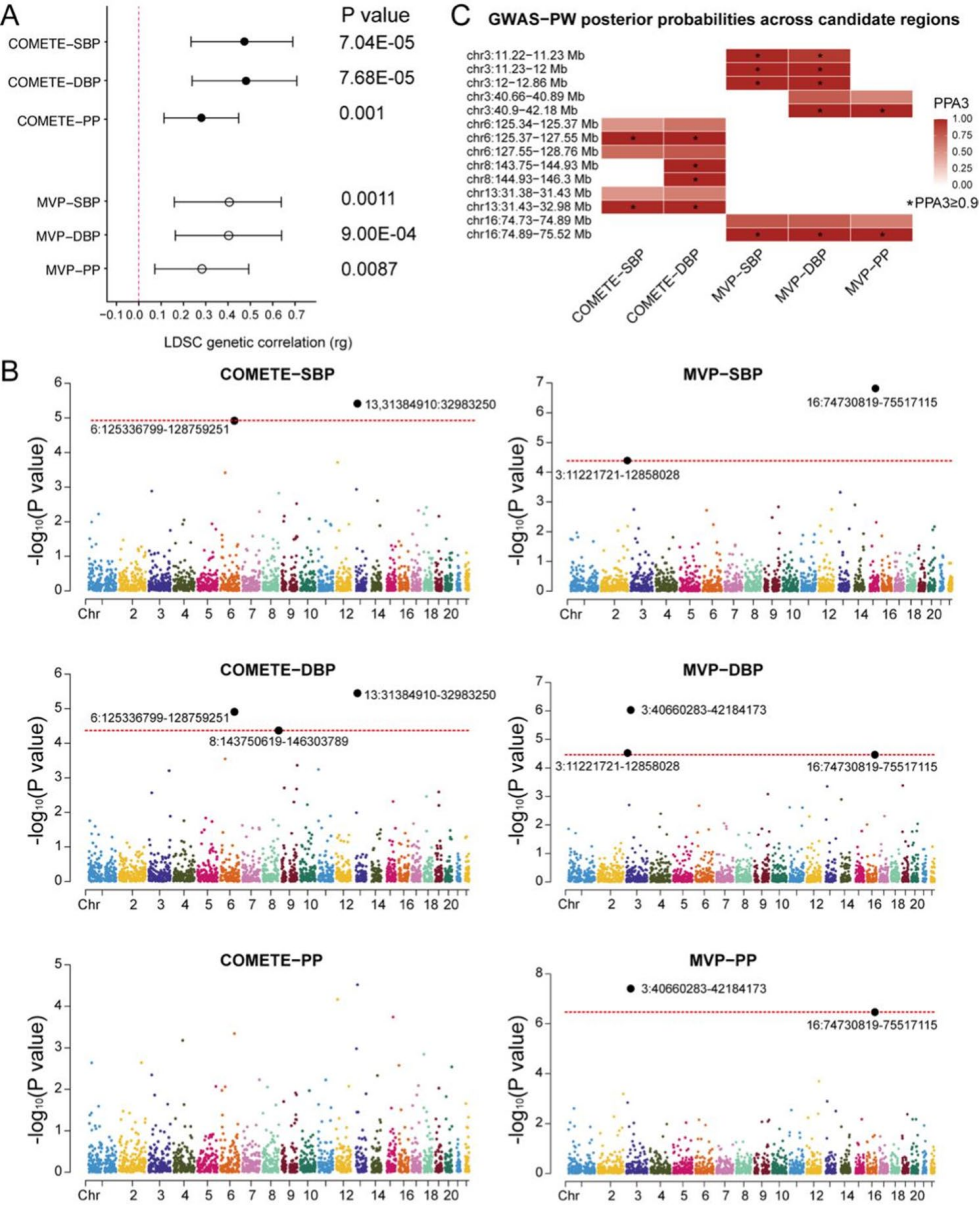
Shared genetic architecture between primary aldosteronism and blood pressure traits. **A**, LDSC genetic correlations between PA and SBP/DBP/PP in COMETE and MVP cohorts. **B**, Manhattan-style plots of SUPERGNOVA local genetic correlation results across six PA–blood pressure comparisons. Significant regions are highlighted by black dots and annotated with genomic coordinates. **C**, GWAS-PW posterior probabilities for candidate regions identified by SUPERGNOVA. Heatmap colours indicate PPA3, and asterisks mark loci with PPA3 ≥ 0.9. COMETE, COrtico et MEdullo-surrénale, les Tumeurs Endocrines; DBP, diastolic blood pressure; GWAS, genome-wide association study; GWAS-PW, Pairwise GWAS; LDSC, Linkage Disequilibrium Score Regression; MVP, Million Veteran Program; PA, primary aldosteronism; PP, pulse pressure; SBP, systolic blood pressure

We first assessed genome-wide genetic correlations between PA and three BP phenotypes (SBP, DBP and PP) using COMETE as the discovery cohort and MVP as the validation cohort (Additional file 1: Table S1). In the COMETE cohort, PA showed significant positive genetic correlations with all BP traits (SBP: rg = 0.462 ± 0.116, *P* = 7.0 × 10^− 5^; DBP: rg = 0.474 ± 0.120, *P* = 7.7 × 10^− 5^; PP: rg = 0.280 ± 0.085, *P* = 0.0010). The MVP cohort yielded concordant results (SBP: rg = 0.398 ± 0.122, *P* = 0.0011; DBP: rg = 0.402 ± 0.122, *P* = 9.0 × 10^− 4^; PP: rg = 0.282 ± 0.107, *P* = 0.0087), indicating a reproducible direction of genetic correlation with broadly consistent magnitudes across independent PA datasets (Fig. 2A). Cross-trait intercepts were close to zero, suggesting negligible sample overlap or confounding inflation (Additional file 1: Table S2). GNOVA analysis produced comparable estimates, further supporting the robustness of the observed PA-BP genetic overlap (Additional file 1: Table S2). Together, these findings indicate that PA and BP traits share a reproducible polygenic component across independent cohorts.

### Local genetic correlation identifies discrete regional PA–BP overlap

We next assessed whether genome-wide PA–BP genetic overlap was concentrated within specific genomic regions using SUPERGNOVA. Across the COMETE and MVP cohorts, 13,944 linkage disequilibrium blocks were examined, of which 324 showed nominal evidence of local genetic correlation (*P* < 0.05; Additional file 1: Table S3). After Bonferroni correction, 12 tests across five PA-BP trait pairs remained significant: COMETE-SBP, COMETE-DBP, MVP-SBP, MVP-DBP and MVP-PP, whereas COMETE-PP yielded no significant loci (Additional file 1: Table S4). After merging overlapping linkage disequilibrium blocks, these signals corresponded to six independent genomic regions (Fig. 2B). Several regions, including chr6:125.3–128.8 Mb and chr13:31.3–32.9 Mb in the COMETE cohort, showed local correlations with both SBP and DBP. These findings suggest that selected genomic regions contribute disproportionately to the shared PA-BP genetic signal, although most of the overlap remains broadly polygenic. Significant regions accounted for approximately 12.7% of the total genome-wide polygenic covariance at the nominal threshold and 1.0% after FDR correction. To assess whether locally correlated regions were also consistent with shared association signals, we applied GWAS-PW. This complementary Bayesian approach identified nine genomic loci with high posterior probabilities of shared association (PPA3 ≥ 0.9; Fig. 2C; Additional file 1: Table S5), consistent with the SUPERGNOVA results. These findings support the presence of discrete regional signals contributing to the shared genetic architecture between PA and BP traits.

### Cross-trait meta-analysis identifies shared PA–BP loci enriched in regulatory and tissue-specific contexts

Given the reproducible genetic correlations between PA and BP traits, we next applied MTAG and CPASSOC as complementary cross-trait meta-analysis methods to identify variants associated with both phenotypes. After linkage disequilibrium pruning at genome-wide significance (*P* < 5 × 10^− 8^), 4,889 genome-wide significant SNPs were jointly detected. Co-localisation analysis was then performed at loci identified by cross-trait meta-analysis, identifying 143 independent variants (2.9%) with evidence consistent with a shared association signal (PP.H4 ≥ 0.8; Additional file 1: Table S6; Additional file 2: Fig S1). For all 143 shared variants, PP.H4 exceeded the posterior probabilities for the PA-only, BP-only and distinct-association models, supporting a shared-association model rather than a purely BP-only model. To further assess whether these signals were primarily driven by the larger BP GWAS, we examined PA-side association evidence in the original PA GWAS datasets. Overall, 36 of the 143 co-localised variants showed nominal association with PA in at least one original PA GWAS dataset, including 28 variants in COMETE and 28 variants in MVP, with 20 variants supported in both datasets (Additional file 1: Table S6). Nevertheless, because only a subset of signals showed nominal PA-side support in the original PA GWAS datasets and the BP GWAS sample sizes were substantially larger, we interpreted these loci conservatively as PA-BP shared association signals rather than definitive PA-specific susceptibility loci.

**Fig. 3:**
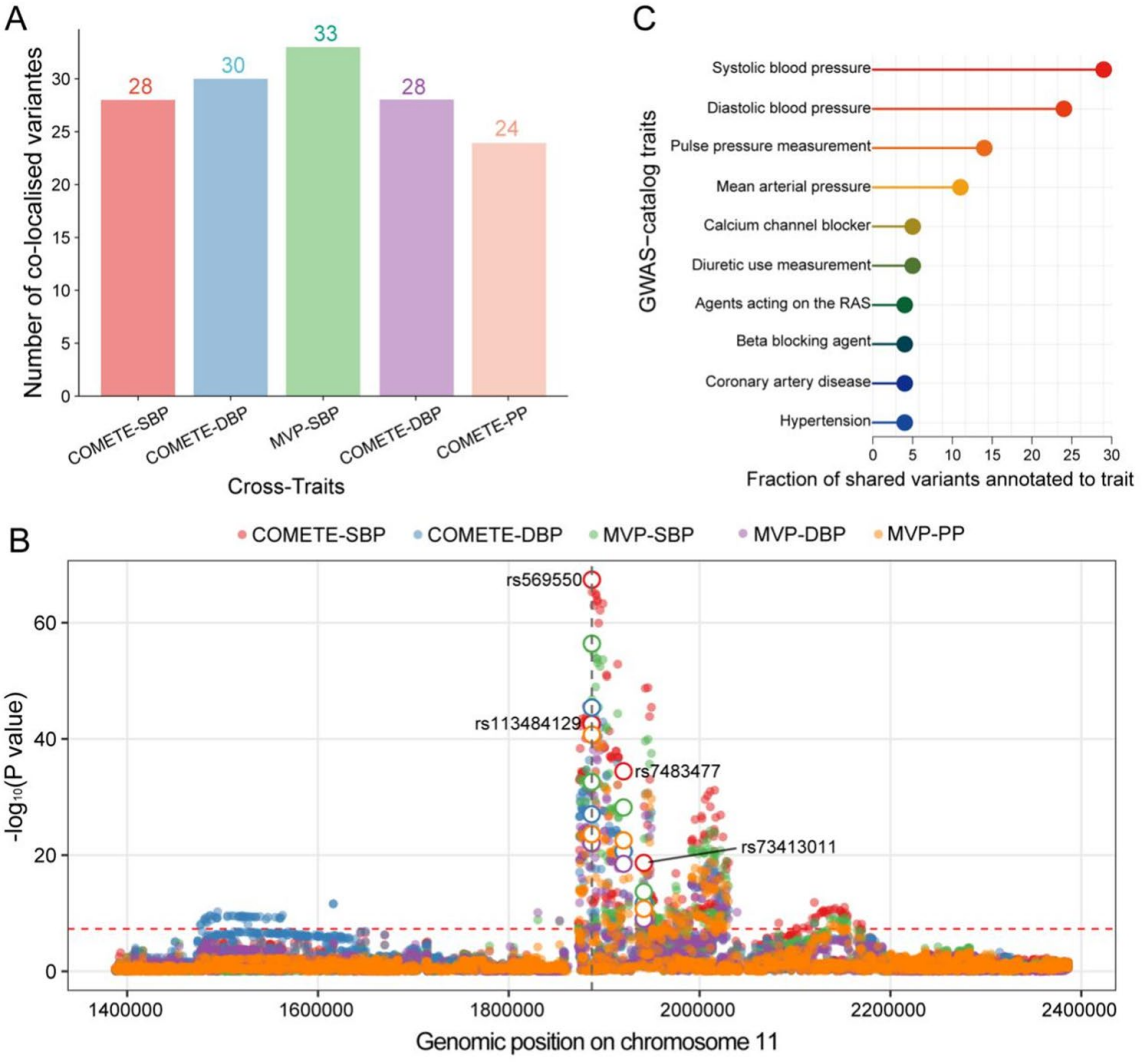
Cross-trait meta-analysis identifies shared PA-BP association signals. **A**, Bar plot showing the number of independent variants identified by cross-trait meta-analysis and supported by co-localisation analysis across PA-BP trait pairs in the COMETE and MVP cohorts. **B**, Regional association plot at the chromosome 11p15 locus highlighting recurrent PA-BP association signals across COMETE and MVP trait pairs. Each point represents a variant, colour-coded by PA-BP trait pair. The y-axis shows –log10(*P* value), and the x-axis indicates genomic position. Lead recurrent variants are annotated. **C**, GWAS Catalog trait annotation of shared PA-BP variants, showing the proportion of variants overlapping previously reported trait associations. COLOC, co-localisation analysis; COMETE, COrtico et MEdullo-surrénale, les Tumeurs Endocrines; DBP, diastolic blood pressure; GWAS, genome-wide association study; MVP, Million Veteran Program; PA, primary aldosteronism; PP, pulse pressure; SBP, systolic blood pressure

The number of co-localised variants was broadly consistent across the five PA-BP trait pairs (Fig. 3A), and 33 variants were observed across more than one trait pair (Additional file 1: Table S7). Four variants, rs113484129, rs569550, rs7483477 and rs73413011, were detected in all five PA-BP combinations and mapped to the chromosome 11p15 region (Fig. 3B; Additional file 1: Table S7). These recurrent signals suggest that a subset of genomic regions contributes consistently to the shared genetic architecture of PA and BP across haemodynamic phenotypes.

Functional annotation using ANNOVAR showed that, among the 143 co-localised variants, 58 (40.6%) were intronic and 40 (28.0%) were intergenic (Additional file 1: Table S6). Four variants (2.8%) had CADD scores > 12.37 and were therefore predicted to have potential functional relevance: rs34869093 in the COMETE–SBP pair, nearest gene *HNRNPA1L3*; rs2128416 in the COMETE–SBP pair, nearest gene *CASZ1*; rs706007 in the COMETE–DBP and MVP–DBP pairs, nearest gene *CASZ1*; and rs75248620 in the COMETE–DBP pair, nearest gene *SBF2* (Additional file 1: Table S6). These loci include genes previously implicated in cellular stress responses, vascular development and ion-transport-related biology, supporting their relevance for downstream prioritisation.

Constraint analysis using LOEUF identified 20 variants mapping to genes or genomic regions with high intolerance to loss-of-function variation (LOEUF < 0.6; Additional file 1: Table S6). These included loci proximal to *CASZ1*, *SH3PXD2A*, *TBPL1* and *SBF2*, several of which were detected across multiple PA–BP pairs. In particular, rs706007, located near *CASZ1* and associated with a low LOEUF score (0.285), was observed in both PA cohorts, supporting its prioritisation as a recurrent cross-trait signal.

Evolutionary conservation analysis using PhastCons and PhyloP showed that most shared variants occurred in regions with limited cross-species conservation, with 68.5% exhibiting PhastCons = 0 and 72.0% showing negative PhyloP values (Additional file 1: Table S6). This pattern is consistent with a substantial contribution from regulatory regions that may be evolutionarily flexible or lineage-specific. However, three variants showed high PhastCons scores: rs34869093 = 1.00, rs75248620 = 0.996 and rs706007 = 0.965. Two variants, rs75248620 and rs34869093, also had PhyloP values > 2, indicating stronger positional conservation. Together with the constraint analysis, these findings highlight a subset of shared variants with convergent functional-prioritisation evidence.

Tissue annotation using snpXplorer showed transcriptional activity of shared variants in adrenal gland, arterial tissues, heart and adipose tissue, consistent with tissues relevant to PA and BP biology (Additional file 1: Table S6). Additional annotations in hypothalamic and pituitary regions suggested possible involvement of neuroendocrine regulatory contexts. To further contextualise these findings, we examined overlap between shared PA-BP variants and previously reported GWAS traits. Most overlapping associations were related to BP, vascular and cardiovascular phenotypes (Fig. 3C). Together, these analyses indicate that shared PA-BP variants are enriched in regulatory and trait contexts relevant to cardiovascular and BP biology, while remaining consistent with a polygenic and multi-tissue model of BP regulation.

### Gene-level prioritisation identifies recurrent PA-BP candidate genes

**Fig. 4:**
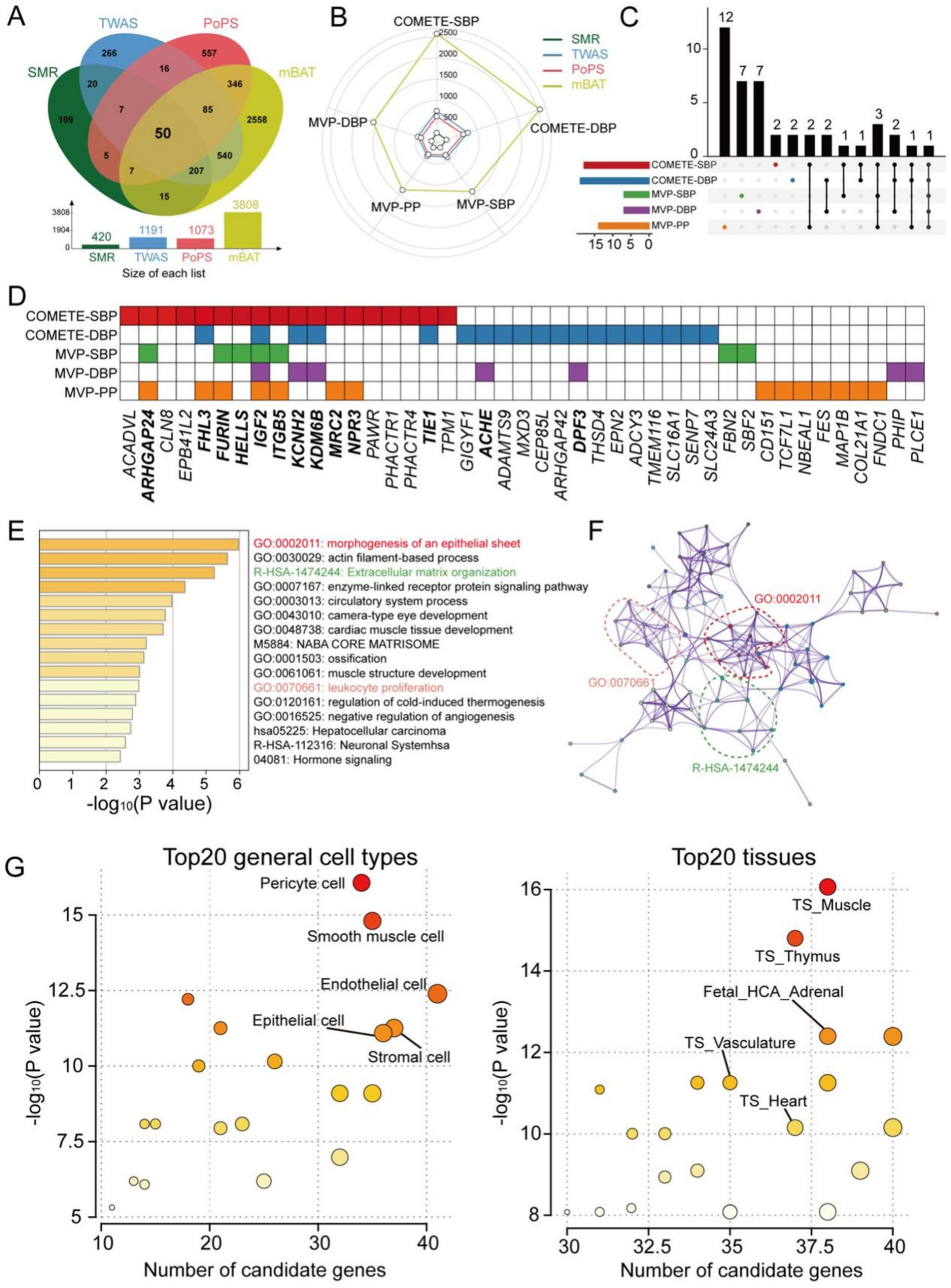
Gene-level prioritisation and functional annotation of PA–BP candidate genes. **A**, Venn diagram showing the overlap of candidate genes identified by four complementary gene-prioritisation approaches: SMR, TWAS, PoPS and mBAT-combo. Numbers indicate genes identified by each method and their intersections. **B**, Radar plot summarising the number of candidate genes identified for each PA–BP trait pair across the COMETE and MVP cohorts using different gene-based methods. **C**, UpSet plot showing overlap of candidate genes across PA–BP trait combinations, highlighting genes shared by multiple trait pairs. **D**, Heatmap showing the presence of prioritised PA–BP candidate genes across trait pairs. **E**, Functional enrichment analysis of prioritised candidate genes. The bar plot shows enriched Gene Ontology (GO) terms and Reactome pathways ranked by −log10(*P* value). **F**, Network representation of enriched functional pathways derived from prioritised candidate genes. Nodes represent enriched GO or Reactome terms and edges indicate shared genes between pathways. **G**, Cell- and tissue-specific enrichment analysis of prioritised PA–BP candidate genes, highlighting enrichment across vascular, perivascular, stromal-associated and adrenal-relevant cellular contexts. BP, blood pressure; COMETE, COrtico et MEdullo-surrénale, les Tumeurs Endocrines; DBP, diastolic blood pressure; GO, Gene Ontology; GWAS, genome-wide association study; mBAT-combo, Multi-Trait Gene-Based Association Test; MVP, Million Veteran Program; PA, primary aldosteronism; PoPS, Polygenic Priority Score; PP, pulse pressure; SBP, systolic blood pressure; SMR, Summary-data-based Mendelian Randomisation; TWAS, transcriptome-wide association study

Assigning GWAS variants to genes solely based on genomic proximity may overlook regulatory and pleiotropic effects. To refine gene-level associations, we integrated four complementary approaches: SMR, TWAS, PoPS and mBAT-combo. These analyses identified 420, 1,191, 1,073 and 3,808 significant genes, respectively (Additional file 1: Table S8-11). Fifty genes were consistently identified across all four methods (Fig. 4A and B), of which 43 were linked to at least one PA-BP trait pair (Fig. 4C; Additional file 1: Table S12). Twelve genes showed consistent evidence across both PA cohorts, supporting cross-cohort reproducibility (Fig. 4C). Among these, *IGF2* was the most recurrent candidate gene, reaching significance across all five PA–BP combinations. Additional genes, including *ARHGAP24*, *FURIN*, *ITGB5*, *KCNH2*, *KDM6B*, *ACHE*, *DPF3*, *MRC2*, *NPR3*, *HELLS*, *FHL3* and *TIE1*, were significant in at least two PA–BP pairs (Fig. 4D). Six of these recurrent genes (*IGF2*, *ARHGAP24*, *ITGB5*, *KCNH2*, *DPF3* and *NPR3*) were also identified in our previous transcriptomic study of sodium-induced remodelling of the pig adrenal cortex, where they showed differential expression under high- versus low-salt dietary conditions [50]. This cross-cohort and cross-species concordance supports their biological relevance in contexts related to adrenal function, salt responsiveness and BP regulation, while not establishing direct mechanistic causality.

Functional enrichment analysis showed that the prioritised genes were enriched for biological processes related to morphogenesis of an epithelial sheet, extracellular matrix organisation, circulatory system processes and leukocyte proliferation (Fig. 4E and F; Additional file 1: Table S13). These findings suggest that shared PA-BP candidate genes converge on pathways relevant to tissue remodelling, vascular biology and immune-associated processes.

Using the GENE2FUNC function in FUMA, we found that 26 PA-BP candidate genes were significantly enriched for haemodynamic traits, including SBP, DBP, mean arterial pressure, hypertension and PP, which represented the most strongly associated trait categories (Additional file 1: Table S14; Additional file 2: Fig S2). Additional enrichments were observed for extracellular matrix and elastic fibre organisation, as well as traits related to bone mineral density, body fat mass and metabolic regulation. These associations are consistent with broader cardiovascular and metabolic contexts of aldosterone biology, although they should be interpreted as annotation-based links rather than direct evidence of disease mechanisms [54].

Cell- and tissue-specific enrichment analysis of the 43 PA-BP candidate genes showed strongest enrichment in vascular and perivascular cellular contexts, including pericytes, smooth muscle cells and endothelial-related categories (Fig. 4G). Tissue-level analyses showed broader enrichment across blood vessel, nerve, adipose and adrenal-associated signatures, with additional cell-specific enrichment in neural and stromal-associated signatures (Additional file 2: Fig. S3 and S4). These expression patterns place PA-BP candidate genes within vascular, metabolic and endocrine-relevant tissue contexts, supporting a multi-tissue model of shared genetic susceptibility.

### *SLC24A3* is enriched in aldosterone-producing cell states in APA

**Fig. 5:**
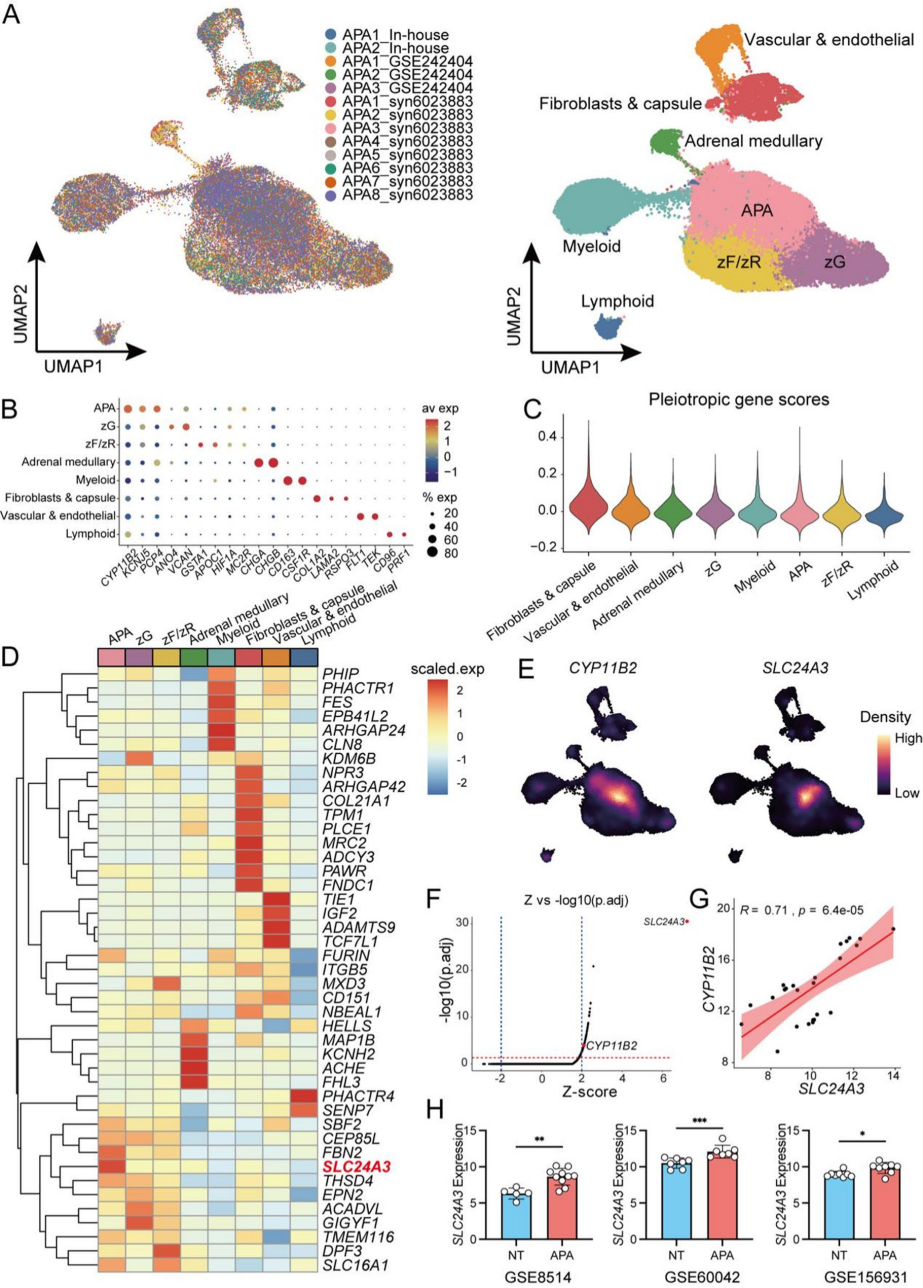
Prioritised PA-BP candidate genes map to adrenal cell states, with SLC24A3 enriched in aldosterone-producing populations. **A**, UMAP visualisation of integrated single-cell and single-nucleus RNA-seq data from APA samples, coloured by sample source (left) and annotated cell type (right). **B**, Dot plot showing expression of canonical marker genes across annotated cell types, with dot size indicating the percentage of expressing cells and colour representing average expression. **C**, Violin plots showing module scores for the 43 prioritised PA–BP candidate genes across major adrenal cell populations. **D**, Heatmap showing scaled expression of prioritised PA-BP candidate genes across annotated cell types. **E**, UMAP visualisation of kernel density estimates for *CYP11B2* and *SLC24A3* expression across cell states. **F**, Exploratory in silico perturbation analysis of *SLC24A3* using scTenifoldKnk, showing genes with altered inferred regulatory connectivity after simulated *SLC24A3* perturbation. Genes are ranked by Z scores derived from network perturbation statistics, reflecting the magnitude and statistical support for altered network connectivity rather than directional changes in gene expression. **G**, Association between *SLC24A3* and *CYP11B2* expression in APA samples from bulk transcriptomic analysis across three independent RNA-seq datasets. **H**, Differential expression of *SLC24A3* between APA and non-tumoural adrenal cortex (NT) tissues. APA, aldosterone-producing adenoma; BP, blood pressure; CYP11B2, cytochrome P450 family 11 subfamily B member 2 (aldosterone synthase); NT, non-tumoural adrenal cortex; PA, primary aldosteronism; SLC24A3, Solute Carrier Family 24 Member 3; UMAP, uniform manifold approximation and projection; zF, zona fasciculata; zG, zona glomerulosa; zR, zona reticularis. *P < 0.05, **P < 0.01, ***P < 0.001

To define the adrenal cellular context of prioritised PA-BP candidate genes, we integrated 13 APA samples from two independent single-cell or single-nucleus APA cohorts with our previously generated APA datasets. After batch-effect correction, dimensionality reduction, clustering and cell-type annotation, we constructed a unified cellular atlas of APA and adjacent adrenal tissue (Fig. 5A and B). At the global level, module scores for the 43 prioritised PA-BP candidate genes were enriched in stromal-associated compartments, including fibroblast/capsule cells, vascular cells and endothelial populations (Fig. 5C). These findings are consistent with the tissue- and cell-type enrichment analyses and support a prominent non-parenchymal component of the shared PA-BP genetic signal.

Although the aggregate candidate-gene signal was most prominent in non-parenchymal compartments, we next examined a candidate within the steroidogenic compartment to probe the adrenal-specific component of the shared signal. Among the 43 prioritised genes, we focused on *SLC24A3* as an illustrative steroidogenic-compartment candidate because, among adrenal steroidogenic populations, its expression was highest in APA cells (Fig. 5D), and it encodes a Na^+^/Ca^2+^/K^+^ exchanger with potential relevance to calcium-dependent adrenal steroidogenesis. Within the steroidogenic compartment, *SLC24A3* expression was also detectable in zona glomerulosa cells of adjacent adrenal tissue (Fig. 5D and E). This distribution suggests that *SLC24A3* is associated with aldosterone-producing cell states rather than being strictly tumour-specific. Its expression showed cellular and spatial overlap with *CYP11B2*, supporting further analysis of *SLC24A3* in aldosterone-producing populations.

To explore whether *SLC24A3* was connected to aldosterone-associated transcriptional networks, we performed in silico perturbation analysis using scTenifoldKnk. Simulated perturbation of *SLC24A3* produced network-level changes, with *CYP11B2* among the most affected nodes in the inferred regulatory network (Fig. 5F; Additional file 1: Table S15). However, experimental siRNA-mediated knockdown of *SLC24A3* efficiently reduced *SLC24A3* expression but did not significantly alter *CYP11B2* expression in HAC15 cells, indicating that the computationally inferred association is unlikely to reflect direct regulation of *CYP11B2* under these conditions (Additional file 2: Fig. S5A and B).

Consistent with this interpretation, analysis of three independent bulk RNA-seq cohorts showed that *SLC24A3* expression was positively associated with *CYP11B2* expression (Fig. 5G) and was higher in APA than in non-tumoural or adjacent adrenal tissue (Fig. 5H). Together, these findings identify *SLC24A3* as a PA-BP candidate gene that is enriched in aldosterone-producing adrenal cell states and associated with steroidogenic transcriptional programmes, while not establishing a direct causal role in regulating *CYP11B2* expression.

### Spatial heterogeneity of *SLC24A3* expression in APA

**Fig. 6:**
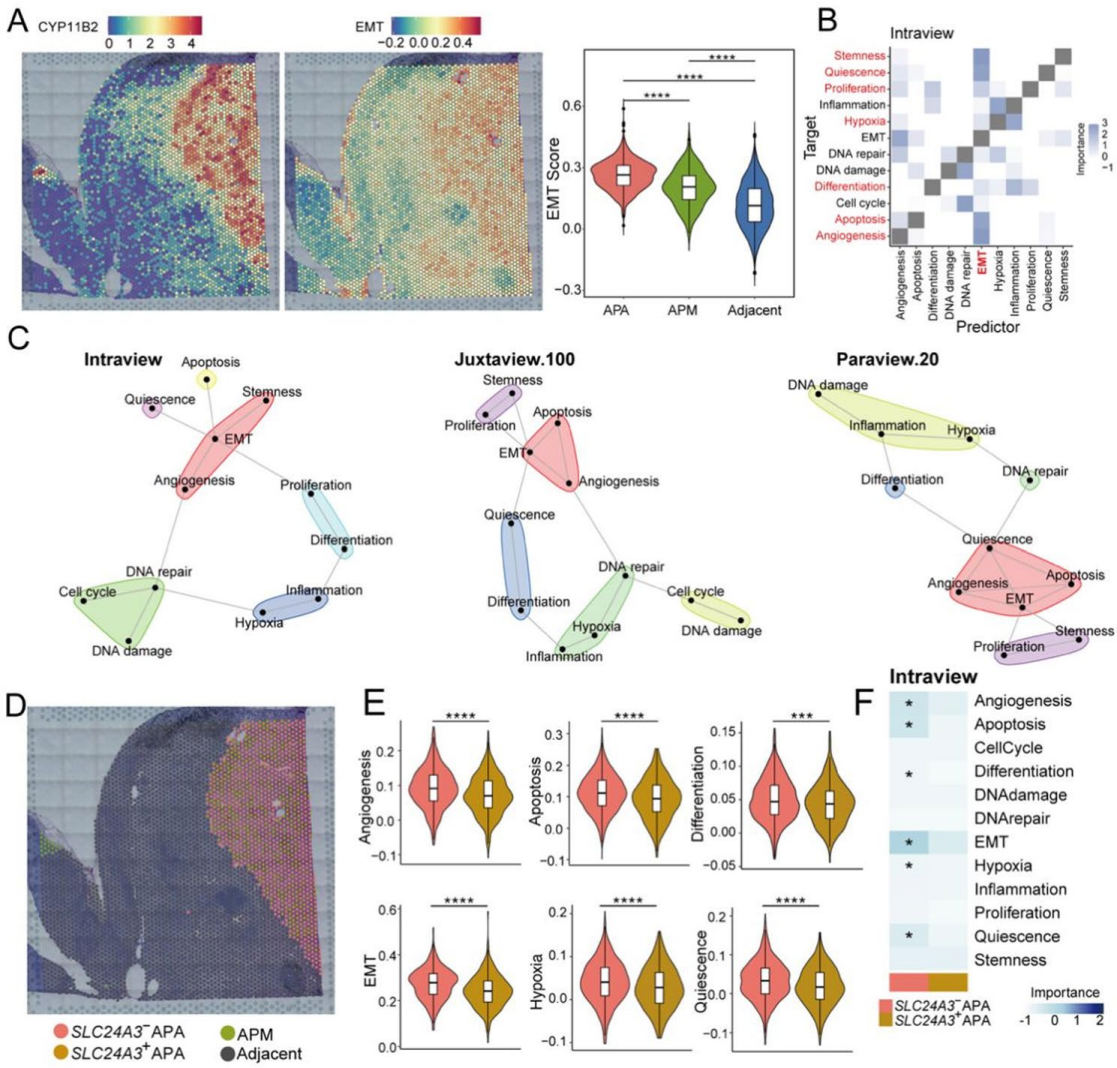
Spatial heterogeneity of *SLC24A3* expression is associated with APA transcriptional state variation. **A**, Spatial transcriptomic maps showing the distribution of *CYP11B2* expression (left) and EMT pathway scores (middle), with violin plots comparing EMT scores among APA, APM and adjacent adrenal regions (right). **B**, Heatmap of Intraview pathway associations inferred by MISTy, showing relative importance scores among CancerSEA-defined transcriptional states. **C**, Network representations of pathway associations across spatial contexts, including Intraview (left), JuxtaView (middle) and ParaView (right). **D**, Spatial annotation of tissue regions stratified into *SLC24A3*⁺ and *SLC24A3*⁻ APA areas, together with APM and adjacent adrenal regions. **E**, Violin plots comparing pathway activity scores between *SLC24A3*⁻ and *SLC24A3*⁺ APA regions, showing differences in angiogenesis, apoptosis, differentiation, EMT, hypoxia and quiescence-associated transcriptional programmes. **F**, Summary heatmap of Intraview relative importance scores showing transcriptional programmes associated with *SLC24A3*-defined APA regions. APA, aldosterone-producing adenoma; APM, aldosterone-producing micronodule; CYP11B2, cytochrome P450 family 11 subfamily B member 2 (aldosterone synthase); EMT, epithelial–mesenchymal transition; FFPE, formalin-fixed paraffin-embedded; *SLC24A3*, solute carrier family 24 member 3; zF, zona fasciculata; zG, zona glomerulosa; zR, zona reticularis. ***P < 0.001, ****P < 0.0001

To characterise the spatial organisation of *SLC24A3* expression within APA regions, we performed spatial transcriptomic analyses and evaluated 12 tumour cell states and pathway signatures curated in the CancerSEA database using the MISTy framework. At the pathway level, epithelial-mesenchymal transition (EMT) scores were higher in APA regions than in non-APA adrenal regions, including aldosterone-producing micronodules (APM) [25] and surrounding adrenal cortex (Fig. 6A). These findings indicate enrichment of EMT-associated transcriptional programmes within tumour regions.

Spatial dependency analysis using MISTy showed that EMT activity in APA was associated with multiple tumour-related transcriptional programmes (Fig. 6B). Across intra-, juxta- and para-view contexts, EMT-associated programmes showed spatial association with signatures related to angiogenesis, apoptosis, stemness, quiescence and proliferation (Fig. 6C). These findings suggest spatial co-organisation of EMT-related transcriptional activity with stress-adaptive and proliferative programmes within the APA microenvironment, rather than direct functional regulation between these pathways [55].

We next examined *SLC24A3* expression within APA tissue. Spatial transcriptomic profiling showed marked intratumoural heterogeneity of *SLC24A3* expression within APA regions. Based on detectable expression, APA tumour areas were stratified into *SLC24A3*-positive (*SLC24A3*⁺ APA) and *SLC24A3*-negative (*SLC24A3*⁻ APA) subregions (Fig. 6D; Additional file 2: Fig. S6A). In line with the single-cell and bulk RNA-seq analyses, *SLC24A3*⁺ regions showed transcriptional features characteristic of aldosterone-producing cell states, including spatial association with steroidogenic gene expression. However, because the present analyses are transcriptomic and spatially associative, *SLC24A3* expression should be interpreted as a marker of steroidogenic transcriptional context rather than evidence of direct regulation of aldosterone biosynthesis. In contrast, *SLC24A3*⁻ APA regions showed higher EMT-associated activity together with quiescence-associated and stress-related transcriptional signatures (Fig. 6E; Additional file 2: Fig. S6B). These findings do not establish functional differences in steroidogenesis directly but suggest that lower *SLC24A3* expression is associated with APA regions showing reduced steroidogenic transcriptional features and increased stress-adaptive programmes [55].

ParaView spatial reconstruction further indicated that the association between *SLC24A3*⁻ APA regions and these transcriptional programmes was most evident within intratumoural regions, where *SLC24A3*⁻ areas showed spatial co-organisation with EMT-, quiescence- and stress-associated signatures (Fig. 6F). These spatial patterns were observed across three independent APA spatial transcriptomic datasets, supporting reproducibility of the spatial association (Additional file 2: Fig. S6C and D).

Collectively, these spatial analyses indicate that *SLC24A3* expression marks heterogeneous transcriptional states within APA tissue, linking aldosterone-producing regions to distinct spatial organisation of tumour-associated programmes.

## Discussion

In this study, we applied an integrative genetic and transcriptomic approach to characterise the shared genetic architecture of PA and BP traits using two independent PA GWAS datasets, COMETE and MVP. We observed reproducible genome-wide genetic correlations between PA and SBP, DBP and PP, identified 143 independent shared variants supported by co-localisation evidence and prioritised 43 candidate genes using convergent gene-based approaches. Functional, evolutionary, tissue-specific and cell-type annotations placed these shared signals within biological contexts relevant to endocrine, vascular and cardiometabolic regulation. Integration with single-cell and spatial transcriptomic datasets further showed that prioritised PA-BP candidate genes map to defined adrenal cell states, including aldosterone-producing populations and stromal-associated compartments. Together, these findings support a shared polygenic architecture between PA and BP and provide a framework for interpreting PA within the broader biology of BP regulation.

Previous observational and mechanistic studies have established that aldosterone excess in PA contributes to hypertension, vascular remodelling, inflammation and increased cardiovascular risk through mechanisms extending beyond volume expansion and renal sodium retention [7, 8, 56]. Our genetic correlation analyses add a complementary population-genetic perspective by showing that PA and BP traits share an inherited component. The correlations were observed across two independent PA datasets and three BP phenotypes, supporting reproducibility of the overall signal. Although the correlations were strongest for systolic and diastolic BP, pulse pressure showed weaker but directionally consistent associations. This pattern is compatible with the recognised contribution of both volume expansion and aldosterone-mediated vasoconstriction to BP elevation in PA [57], suggesting that shared PA-BP susceptibility may relate more closely to pathways influencing intravascular volume and peripheral vascular resistance than to arterial stiffness alone. Importantly, these analyses do not establish causality between PA and BP traits but indicate that part of their clinical association may reflect shared genetic susceptibility in addition to the downstream haemodynamic effects of aldosterone excess. This overlap likely reflects a combination of endocrine pathways, vascular biology and background BP-related genetic contributions.

An important consideration is whether these correlations reflect aldosterone-related genetic susceptibility or simply capture background essential hypertension genetics within the PA cohorts. Several observations argue against a purely generic-hypertension explanation. The shared signals were not distributed uniformly across hypertension-associated genomic space but converged on discrete regions and on genes recoverable across two independently ascertained PA cohorts, several of which were also differentially expressed in the pig adrenal zona glomerulosa under altered dietary sodium [50]. This pattern is more consistent with adrenal-relevant biology than with diffuse background hypertension risk alone. More directly, integration with single-cell and spatial transcriptomic data localised the prioritised genes to defined adrenal compartments, including aldosterone-producing cell states, rather than to ubiquitously expressed or purely vascular programmes. A signal driven solely by background BP genetics would not be expected to show this adrenal cell state specificity. Several recurrent candidate genes also have established roles in adrenal development, steroidogenic regulation or calcium-dependent signalling, providing biological coherence beyond generic vascular tone. These observations do not exclude a contribution from background hypertension susceptibility, which likely forms part of the shared signal, but they suggest that the PA-BP overlap is not reducible to it and maps, at least in part, onto aldosterone-producing adrenal biology.

Regional genetic analyses further suggested that this shared architecture includes both diffuse polygenic components and discrete genomic regions with stronger local overlap. This pattern is consistent with prior GWAS evidence showing that PA risk signals are concentrated within selected chromosomal regions rather than being uniformly distributed across the genome [15]. Cross-trait meta-analysis and co-localisation identified recurrent PA-BP signals, including four variants present across all five PA-BP trait combinations within a linkage disequilibrium block on chromosome 11p15 encompassing the *LSP1-TNNT3* region. This locus has been implicated in BP regulation and is enriched in arterial regulatory annotations in large BP GWAS datasets [12, 58]. *LSP1* has also been associated with PA and resistant hypertension [15, 16], and encodes a cytoskeletal protein involved in leukocyte motility and endothelial biology [59]. Although the precise causal gene and mechanism at this locus remain unresolved, these findings are consistent with the relevance of vascular and inflammatory regulatory contexts to the shared PA-BP genetic signal.

Several additional shared variants showed convergent evidence from conservation and constraint analyses. For example, rs706007 is located within *CASZ1*, a locus previously implicated in BP regulation and PA [12, 15], and encoding a transcription factor with established roles in vascular assembly and morphogenesis [60]. Similarly, rs75248620 maps to the *SBF2* locus, which encodes a myotubularin-related pseudophosphatase involved in vesicle trafficking and endolysosomal dynamics [61]. *SBF2* was also recovered in the gene-based analyses, providing consistency between variant-level and gene-level prioritisation. These observations do not define causal mechanisms but highlight candidate loci in which multiple lines of evidence converge and which may warrant further functional investigation.

Beyond variant-level signals, our gene-based analyses identified 43 candidate genes supported across SMR, TWAS/FUSION, PoPS and mBAT-combo. This convergence suggests that shared PA-BP susceptibility can be mapped to transcriptionally and functionally annotated genes, rather than being interpreted solely through nearest-gene assignment. Several recurrent genes have established relevance to cardiovascular, endocrine or ion-regulatory biology. *IGF2*, which was identified across all five PA-BP trait pairs, has roles in adrenal development, vascular remodelling and cardiac hypertrophy [62–64]. Other prioritised genes, including *FURIN*, *NPR3*, *ITGB5* and *KCNH2*, are linked to renin-angiotensin system biology, vascular tone, fluid balance, cytoskeletal remodelling and ion-channel signalling [65–68]. These annotations support biological plausibility for shared endocrine-vascular pathways, while remaining consistent with a polygenic model in which no single gene is expected to explain the PA–BP relationship.

Integration of genetic findings with adrenal single-cell and spatial transcriptomic data enabled the prioritised PA-BP candidate genes to be placed within tissue context. This showed enrichment in stromal-associated, vascular and endothelial compartments, consistent with tissue-enrichment results implicating vascular and perivascular cell types. This is relevant because PA is not only a disorder of aldosterone-producing cells, but also involves vascular, immune, and extracellular matrix-associated processes that may contribute to tissue remodelling and cardiovascular risk [6]. Mapping shared candidate genes to adrenal cortical and non-parenchymal cell states therefore provides biological context for genetic signals that would otherwise remain difficult to interpret from GWAS data alone.

Within this context, *SLC24A3* emerged as an illustrative PA-BP candidate gene rather than a definitive mechanistic driver. *SLC24A3* encodes a Na⁺/Ca²⁺/K⁺ exchanger [69], making it biologically plausible in the context of calcium-dependent adrenal steroidogenesis. Single-cell analyses showed enrichment of *SLC24A3* in aldosterone-producing cell states, particularly within APA cells, while bulk transcriptomic analyses showed a positive association between *SLC24A3* and *CYP11B2* expression. However, siRNA-mediated knockdown of *SLC24A3* did not significantly alter *CYP11B2* expression, indicating that the computationally inferred relationship is unlikely to represent direct, context-independent regulation of *CYP11B2*. These findings support interpretation of *SLC24A3* as a marker or component of steroidogenic transcriptional states, rather than as a proven upstream regulator of aldosterone biosynthesis.

Spatial transcriptomic analyses further showed that *SLC24A3* expression is heterogeneous within APA tissue. *SLC24A3*-positive regions showed transcriptional features consistent with aldosterone-producing cell states, whereas *SLC24A3*-negative regions showed higher EMT-associated, quiescence-associated and stress-related transcriptional signatures. These spatial patterns suggest heterogeneity in APA transcriptional states and align with studies showing that endocrine adenomas contain spatially organised programmes of steroidogenesis, stress adaptation and phenotypic plasticity [53, 67, 70, 71]. However, these analyses are associative and transcriptomic; they do not establish that *SLC24A3*-defined regions represent distinct functional compartments or determine hormone output. Further orthogonal validation will be required to determine whether these spatial transcriptional states correspond to functional differences in steroidogenesis, growth or treatment response.

A major strength of this study is the integration of complementary genetic and transcriptomic datasets to interrogate PA-BP overlap at multiple biological scales. The use of two independent PA GWAS datasets, combined with three BP phenotypes, enabled replication of the direction and magnitude of genetic correlations across cohorts. In addition, the convergence of regional genetic correlation, cross-trait meta-analysis, co-localisation and gene-prioritisation methods provided a systematic basis for identifying shared loci and candidate genes. Mapping these signals to single-cell and spatial transcriptomic data further placed prioritised genes within adrenal cellular and tissue contexts, moving beyond nearest-gene annotation alone.

Several limitations should also be considered. PA GWAS sample sizes remain modest compared with BP GWAS, limiting power for subtype-specific analyses and leaving open the possibility that some shared PA-BP signals may primarily reflect broader BP-related genetic architecture rather than PA-specific susceptibility. Differences in PA ascertainment between COMETE and MVP may have contributed to heterogeneity in cohort-specific signals. In particular, PA cases in MVP were identified using PheCode/ICD-based definitions rather than standardised clinical diagnostic criteria, including biochemical screening and confirmatory testing, which may have introduced phenotype misclassification and diagnostic heterogeneity. The restriction to individuals of European ancestry limits generalisability to other populations. Future studies incorporating multi-ancestry PA GWAS data will be important for identifying ancestry-specific risk loci and assessing the transferability of shared PA-BP signals across diverse populations. In addition, the use of summary-level genetic data restricted causal inference and precluded robust Mendelian randomisation because of the limited number of independent PA-associated instruments. Finally, gene-prioritisation, single-cell and spatial transcriptomic analyses provide biological context for candidate genes but do not establish direct functional mechanisms. Further experimental validation in appropriate adrenal models and larger tissue cohorts will be required to define the roles of individual genes and spatial states.

## Conclusion

In summary, this study demonstrates that PA and BP traits share a polygenic architecture that maps to adrenal endocrine cell states and vascular regulatory programmes within the intrinsic adrenal microenvironment. By integrating cross-trait genetics with single-cell and spatial transcriptomic data, we provide a basis for linking inherited BP-related variation to adrenal cell states and tissue organisation. These findings place PA within the wider genetic landscape of BP regulation and identify shared genetic signals, prioritised genes and cellular contexts for future functional and clinical studies. Beyond advancing biological understanding, these findings provide a potential foundation for future translational studies aimed at refining PA risk stratification, improving recognition of endocrine hypertension within broader hypertensive cohorts and identifying adrenal cell-state programmes relevant to cardiovascular risk prevention.

## Supplementary Information

Below is the link to the electronic supplementary material.

Supplementary Material 1: Additional file 1: Supplementary Tables S1-S15.

Supplementary Material 2: Additional file 2: Supplementary Figures S1-S6.

## Data Availability

All data supporting the findings of this study are available within the article and Supplemental Material. Public GWAS summary statistics and transcriptomic datasets are available from the GWAS Catalogue, ICBP/UK Biobank resources, GEO and Synapse under the following accession numbers: COMETE PA GWAS, GCST90129620; MVP PA GWAS, GCST90475695; SBP GWAS, GCST006624; DBP GWAS, GCST006630; PP GWAS, GCST006629; bulk RNA-seq datasets of APAs, GSE8514, GSE60042, and GSE156931; and snRNA-seq datasets of APAs, syn6023883 and GSE242404. Code used for the main analyses is available in GitHub (https://github.com/TAWilliams-lab/GWAS.git). Additional analysis resources are available from the authors on reasonable request.

## Abbreviations

APA: Aldosterone-producing adenoma
CADD: Combined annotation dependent depletion
CSEA: Cell type-specific enrichment analysis
CPASSOC: Cross-phenotype association analysis
EMT: Epithelial-Mesenchymal Transition
GNOVA: Genetic covariance analyzer
GWAS: Genome-wide association studies
HAC15: Human adrenocortical cell line
LDSC: Linkage disequilibrium score regression
LOEUF: Loss-of-function observed/expected upper bound fraction
mBAT: Multi-trait gene-based association test
MTAG: Multi-trait analysis of GWAS
MVP: Million Veteran Program
PA: Primary aldosteronism
PoPs: Polygenic priority score
SLC24A3: Solute Carrier Family 24 Member 3
SMR: Summary-data-based mendelian randomization
TWAS: Transcriptome-wide association study
zG: Zona glomerulosa
