# Supplementary material for: Cross-trait genetic analysis maps shared polygenic architecture between primary aldosteronism and blood pressure to adrenal cell states
Source: J Transl Med. 2026 Aug 13;24:1049. doi: 10.1186/s12967-026-08819-2 (PMC13474680; doi:10.1186/s12967-026-08819-2)
Supplement: Supplementary file 1 [file 12967_2026_8819_MOESM2_ESM.docx]

**Cross-trait genetic analysis maps shared polygenic architecture between primary aldosteronism and blood pressure to adrenal cell states**

Zhuolun Sun*, Changying Jing*, Yunxi Ji, Nicole Reisch and Tracy Ann Williams^#^

Affiliations: Medizinische Klinik und Poliklinik IV, Klinikum der Universität München, Ludwig-Maximilians-Universität München, München, Germany (Z.S., C.J, Y.J., N.R., T.A.W).

*These authors contributed equally to this work.

^#^Corresponding author:

Medizinische Klinik und Poliklinik IV, Klinikum der Universität München,

LMU München, Ziemssenstr. 5, D-80336 München, Germany.

Tel: +49 89 4400 52137; Fax: +49 89 4400 54428;

Email: Tracy.Williams@med.uni-muenchen.de

**Supplementary Figures­**

Figure S1. Circular representation of co-localised variants shared between primary aldosteronism and blood pressure traits…p.1

Figure S2. Phenotypic associations of pleiotropic genes analysed by FUMA…p.2

Figure S3. Enrichment of pleiotropic genes in tissue-specific DEG signatures identified by CSEA…p.3

Figure S4. Enrichment of pleiotropic genes in cell-specific DEG signatures identified by CSEA…p.4

Figure S5. *SLC24A3* knockdown in human adrenocortical cells…p.5

Figure S6. Spatial distribution of *SLC24A3* expression and associated pathway activities across multiple FFPE aldosterone-producing adenoma samples…p.6

**Figure S1. Circular representation of co-localised variants shared between primary aldosteronism and blood pressure traits.**

The circular network depicts genome-wide significant variants identified by cross-trait meta-analysis and supported by co-localisation analysis across PA–BP trait pairs in the COMETE and MVP cohorts. The central nodes represent the PA GWAS cohorts, connected to systolic blood pressure (SBP), diastolic blood pressure (DBP) and pulse pressure (PP). Peripheral nodes correspond to 143 independent variants with evidence consistent with shared association signals based on COLOC analysis. Colours indicate the corresponding BP trait. COMETE, COrtico et MEdullo-surrénale, les Tumeurs Endocrines; COLOC, co-localisation analysis; DBP, diastolic blood pressure; GWAS, genome-wide association study; MVP, Million Veteran Program; PA, primary aldosteronism; PP, pulse pressure; SBP, systolic blood pressure.

**Figure S2. Phenotypic associations of pleiotropic genes analysed by FUMA.**

Phenotype enrichment analysis of the prioritised pleiotropic genes was performed using the GENE2FUNC module in FUMA. The left red bar plot shows the proportion of overlapping genes mapped to each enriched phenotype-associated gene set, and the middle blue bar plot indicates the corresponding enrichment significance as -log10 adjusted P value. The right panel shows the specific overlapping genes contributing to each enrichment signal, with orange squares indicating the presence of a given gene in the corresponding phenotype-associated gene set. Enriched phenotypes are ranked by statistical significance and include blood pressure-related traits, cardiovascular phenotypes, and additional complex traits, highlighting the broader phenotypic context of the identified pleiotropic genes.

**Figure S3. Enrichment of pleiotropic genes in tissue-specific DEG signatures identified by CSEA.**

The 43 pleiotropic genes were enriched across multiple tissue-specific DEG signatures. The three panels show enrichment in upregulated, downregulated, and both-sided signatures, respectively. Bars represent enrichment significance as -log10(*P* value), with red denoting Bonferroni-significant enrichment and blue denoting non-significant enrichment. These findings provide an overview of the tissue contexts potentially involved in the shared genetic architecture of PA and BP traits. CSEA, Cell type-specific enrichment analysis; DEG, differentially expressed genes.

**Figure S4. Enrichment of pleiotropic genes in cell-specific DEG signatures identified by CSEA.**

The 43 pleiotropic genes were enriched across multiple cell-specific DEG signatures. The three panels show enrichment in upregulated, downregulated, and both-sided signatures, respectively. Bars represent enrichment significance as -log10(*P* value), with red denoting Bonferroni-significant enrichment and blue denoting non-significant enrichment. These findings provide an overview of the cellular contexts potentially involved in the shared genetic architecture of PA and BP traits. CSEA, Cell type-specific enrichment analysis; DEG, differentially expressed genes.

**Figure S5. *SLC24A3* knockdown in human adrenocortical cells.
A**, RT-qPCR analysis showing *SLC24A3* expression after siRNA-mediated knockdown in HAC15 human adrenocortical cells. **B**, RT-qPCR analysis showing *CYP11B2* expression after *SLC24A3* knockdown. *SLC24A3* knockdown reduced *SLC24A3* expression but did not significantly alter *CYP11B2* expression under the conditions tested. CYP11B2, cytochrome P450 family 11 subfamily B member 2 (aldosterone synthase); RT-qPCR, reverse transcription quantitative PCR; siRNA, small interfering RNA; SLC24A3, solute carrier family 24 member 3.

**Figure S6. Spatial distribution of *SLC24A3* expression and associated pathway activities across independent aldosterone-producing adenoma samples.**

Spatial transcriptomic analyses across three formalin-fixed paraffin-embedded (FFPE) APA samples show that intratumoural heterogeneity of *SLC24A3* expression is associated with distinct spatial pathway states. **A**, Spatial transcriptomic map showing the distribution of *SLC24A3* expression in one representative FFPE APA sample. **B**, Spatial maps of CancerSEA pathway activity scores across the same APA tissue section. **C**, Spatial annotation of tissue regions stratified into *SLC24A3*-positive and *SLC24A3*-negative APA areas, together with major adrenal cell populations, shown for the other two FFPE APA samples. **D**, Violin plots comparing CancerSEA pathway activity scores between *SLC24A3*-defined APA regions across all three integrated FFPE spatial transcriptomic samples, highlighting differences in angiogenesis-, apoptosis-, differentiation-, EMT-, hypoxia-, and quiescence-related programmes. APA, aldosterone-producing adenomas; EMT, epithelial-mesenchymal transition; FFPE, formalin-fixed paraffin embedded; SLC24A3, Solute Carrier Family 24 Member 3; zF, zona fasciculata; zG, zona glomerulosa; zR, zona reticularis; **P* < 0.05, ***P* < 0.01.
